# Altering Perceived Context: Transportation Cues Influence Novelty-Induced Context Exploration

**DOI:** 10.3389/fnbeh.2021.714927

**Published:** 2021-07-29

**Authors:** Victoria Nemchek, Laura A. Agee, Cassidy A. Malone, Marissa Raskin, Sydney Seese, Marie H. Monfils

**Affiliations:** ^1^Department of Psychology, The University of Texas at Austin, Austin, TX, United States; ^2^Institute for Neuroscience, The University of Texas at Austin, Austin, TX, United States

**Keywords:** context, exploratory behavior, object play, novelty, retention interval, rat, transportation impacts context

## Abstract

Context is the milieu in which everything occurs. Many research studies consider context, or even explicitly manipulate it; yet it remains challenging to characterize. We know that a context surrounds and influences tasks; however, the boundaries of its influence are difficult to define. In behavioral science, context is often operationalized by the physical environment in which the experiment takes place, and the boundaries of the context are assumed to begin at the entrance to that of the room or apparatus. Experiences during transportation to the testing space have been shown to impact rodent behavior and memory, but transportation’s relationship with novelty and physical environment is not fully understood. The current study explored how familiar vs. novel cues, both within a physical environment and preceding it, impact the perception of a context. We manipulated context on three levels: physical testing environment, object cues within that environment, and transportation cues preceding entrance to the testing environment. We found that novel transportation cues can change rats’ perception of both familiar and novel contexts. The effects of transportation on perceived context may be affected by the length of the retention interval, testing environment, and behavioral range. These data suggest that context is a broad concept that includes cues across time and is sensitive to small differences in experience.

## Introduction

Context is often mentioned, and even explicitly manipulated, in many research studies; yet it is rarely comprehensively defined. We know that a context surrounds and influences a task (Rosas et al., 2013); however, the boundaries of its influence are difficult to determine. Which stimuli make up a context and which are contained within it? How far does a context extend in time and space? In behavioral science, context is often operationalized as the physical environment in which the experiment takes place (e.g., the room or apparatus) and the boundaries of the context are assumed to begin at the entrance to that environment in both time and space. Manipulations of the physical testing environment (e.g., changes to flooring, lighting, or scent) are practical to carry out and are therefore common manipulations of context. While such manipulations have been used to demonstrate the importance of context in memory (Bouton and King, 1983; Wilson et al., 2013; Arias et al., 2015), the effects of shifting physical environments are far from uniform. When conditioned cues are presented in a different context than training, the reinforcement is associated with the novel context but not the familiar cue until later in training when the combination of cue and environment is no longer novel. This suggests that memories may become more context-dependent when there is an added element of surprise or novelty (León et al., 2011). Novelty may also play a role in delineating cues from context. Novel stimuli are more salient and are more likely to be treated as a predictive cue (Mackintosh, 1973). Otherwise, familiar cues can fall into the “background” of the context (Nadel and Willner, 1980). The time between events is also well known to impact memory. In the novel object recognition literature, the delay between exposure to familiar objects and the test where a novel object is introduced (the retention period or test delay) impacts rodents’ ability to distinguish between familiar and novel objects (Ennaceur and Delacour, 1988). Therefore, the length of a context in time is likely limited by memory and factors that influence memory.

While physical space—and the exteroceptive cues within that space—is the most commonly manipulated modality in experimental designs aimed at studying context effects, there are many other components that make up a context. Other well-studied contextual modalities are defined by interoceptive stimuli (Bouton, 2018) including hunger (Davidson, 1993; Schepers and Bouton, 2017), drug state (Lattal, 2007), and task demands (Smith and Mizumori, 2006). Experiences even during transportation to the testing space have also been shown to impact rodent behavior and memory (Bevins et al., 2000). Bevins and colleagues showed that when rats were transferred by being carried on an arm to an operant chamber where they had previously experienced a shock, they showed less fear behavior (i.e., freezing) than when transported using a cart as they were for fear conditioning previously. These results suggest that the rats had conditioned—at least in part—to the original transportation cues. Replacement of the original transportation cues with different transport cues was sufficient to reduce context freezing. It is not clear, however, if these rats learned to fear a context that was made up of both the physical environment and the set of transportation cues preceding that environment or if transportation cues were simply predictive of shock.

### Current Study

The current study asks: how do familiar vs. novel cues, both within a physical environment and preceding it, impact contextual recognition? To examine rats’ recognition of a context we utilized a modified novel object design and measured object exploration relating to changes in the environment, the object within that environment, and the transportation preceding entrance to the environment. We found that novel transportation disrupted context recognition and that this effect was modulated by testing delay, context familiarity, and behavioral range. The first study utilized a short retention interval of 1 h and was carried out in the familiar context, the second experiment showed that the effects of transportation are maintained with a long retention interval of 24 h and in a novel context, the last experiment (anchored object) showed that object play modulates the effects of novel transportation.

## Materials and Methods

### Experimental Design

**Figure 1:**
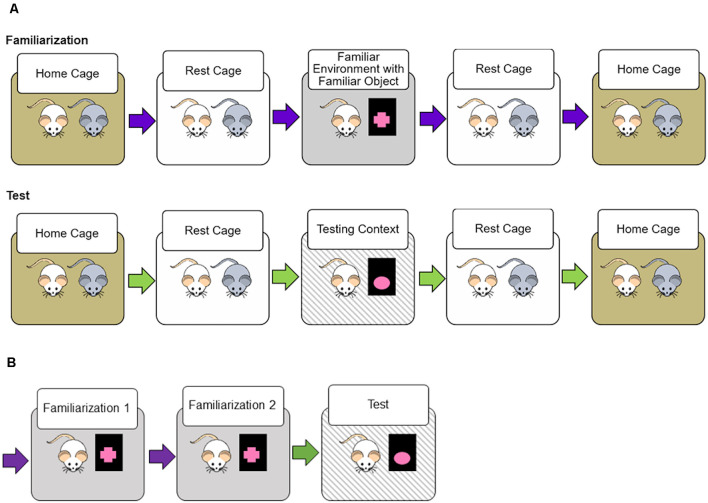
Novel transportation procedure. **(A)** For two 10-min familiarization sessions rats were transported from their homecage to a rest environment with their cage mate. After a 5-min rest period, a single rat is transported to the familiarization context (familiar environment with a familiar object placed within). After the first rat in the pair has habituated for 5 min, the cage mate is then introduced to the familiarization context. Twenty-four hs after the second familiarization session the testing session occurs. Like before, both rats are removed from a homecage and placed in the same rest environment before entrance to the testing context. The testing context was either identical to the familiarization context, or a novel object, novel transportation cues, or both were introduced. **(B)** In each study, rats underwent two familiarization sessions spaced 24 h apart, followed by a 3-min testing session. In the short retention interval study, the delay between the second familiarization session was 1 h, for the long retention interval and anchored object studies the delay was 24 h. Different color arrows depict different transportation cues. All familiarization utilized familiar transportation cues.

The Novel Transportation paradigm extends the logic of Novel Object Recognition tasks: less time spent exploring the object indicates that the subject retained object recognition memory (Ennaceur and Delacour, 1988). We used object exploration as an indirect measure of context familiarity; in a more familiar environment rats should explore the environment less and the object more. Figure 1 demonstrates the Novel Transportation design. Rats undergo 2 days of familiarization where they are removed from their homecage with their cage mate, rest in a separate environment, and are allowed to explore the familiar environment and familiar object for 10 min before removal to the rest cage. Testing is conducted in a similar fashion; object, environment, and transportation could be either familiar or novel during the 3-min testing period.

### Subjects

A total of 166 (short retention interval study: *n* = 24; long retention interval study: *n* = 96; anchored object study: *n* = 46) male Sprague-Dawley rats between 9 and 11 weeks old obtained from Envigo (Houston, TX). Rats were dual housed in clear plastic cages (27 × 48 × 20 cm) on a 12-h light-dark cycle. All experiments took place in the dark cycle. Food (standard rodent chow) and water were provided *ad libitum*. All procedures were conducted under the approval of the Institutional Animal Care and Use Committee at the University of Texas at Austin and in accordance with National Institutes of Health guidelines.

### Transportation

#### Familiar Transportation

We chose variables that would influence a variety of sensory modalities that could change from day to day in a lab. During the familiarization period, and for those exposed to familiar transportation before testing, rats were transported between cages and testing environments rolled on a cart, covered in a dark shroud, in a cage with clean bedding by a single experimenter who wore their hair up and a lab coat.

#### Novel Transportation

Rats exposed to the novel transportation context were carried between testing environments, un-covered, in a cage with no bedding by a pair of experimenters (who conversed during transportation) wearing hair down, a face mask, and scrubs. These variables were counterbalanced across several cohorts of rats.

### Objects

#### Light Objects

**Figure 2:**
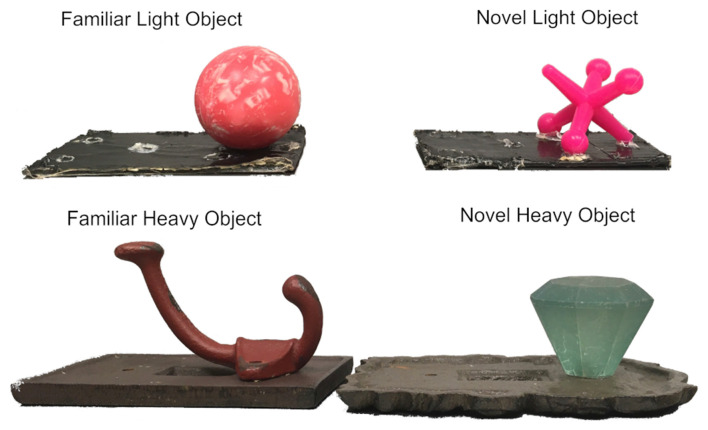
Experiment 1 used light objects, experiment 2 used heavy objects. Light objects measured approximately 8 cm long × 4 cm wide × 3 cm high and weighed 7–30 g. Heavy objects measured approximately 13 cm long × 9.5 cm wide × 5 cm high and weighed 376–427 g. Familiar and novel objects were counterbalanced.

Object 1 was a pink plastic jax on a black plastic base (approximately 8 cm long × 4 cm wide), three objects were used interchangeably. The approximate weight was 8.0 g. Object 2 was a pink rubber ball affixed to plastic bases of the same dimensions. While the jax and the ball were approximately the same diameter (3 cm), the ball was heavier. Object 2 weighed about 29.5 g (see top panel Figure 2).

#### Heavy Objects

Object 1 was a blue glass hexagonal doorknob on dark brown cast iron base 13 cm long × 9.5 cm wide × 5 cm high weighing approximately 380.8 g. Object 2 was a red cast iron hook on dark brown cast iron base 11.5 cm long × 7 cm wide × 6.5 cm high and weighing approximately 426.5 g (see bottom panel Figure 2).

### Environments

#### Familiar: Homecage

The familiar environment was the homecage for each rat, with testing occurring in the colony room. This was chosen for two reasons: (1) to make the familiar environment as familiar as possible; and (2) to allow us to compare environments that were in completely different spaces so that the contexts were as distinct as possible. Since it was deemed best practice to pair-house the rats in these studies, both rats had to be removed from their homecage for it to be used for testing. Objects were added to the homecage after both rats were removed, and different objects were used for each cagemate. Additionally, in order to film the testing sessions, cages were placed on an empty shelf in the same colony room. Both the removal of the cagemate and relocation of the homecage were utilized during the familiarization phase, so these altered components were not novel during testing. Digital cameras were used to record behavior.

#### Novel: Operant Chamber

Novel environments were a standard conditioning chamber equipped with two clear plexiglass walls and two metal walls, stainless steel rod floors, and red lighting and were enclosed in acoustic isolation boxes (Coulbourn Instruments) were used as novel environments. The environment was lit with a red LED during testing and was cleaned with Windex between each testing session. Behavior was recorded with a digital camera mounted in the ceiling of each chamber.

### Behavioral Scoring

The main behavioral measure used was the exploration of the object. Object exploration was defined using the traditional definition from the novel object literature: object exploration involves orienting the snout towards the object at a distance equal to or less than 2 cm, sniffing, licking, biting, or otherwise touching the object were included in object exploration; sitting, standing, or walking on the object were not considered object exploration (Ennaceur and Delacour, 1988). Based on novel object literature we know that exploration can be used to measure relative novelty, in this study we are ultimately interested in context novelty, but traditional definitions of object exploration could not be readily applied to environment exploration. Non-exploratory behaviors were defined as any behavior that is clearly neither exploration of either the object nor the surrounding environment, such behaviors included grooming and sleeping. While some stereotypical fear-induced freezing occurred, it was always accompanied by head scanning movements and was therefore categorized as context exploration. To assess context exploration, the total duration of object exploration and non-exploratory behaviors were subtracted from the total duration of the observation. Therefore, every second of the observation period was accounted for: if a rat was engaged in neither object exploration, nor non-exploratory behaviors the rat must be exploring the context. Using this definition, rats that were sniffing outside of the 2 cm perimeter of the object (including the walls and lid of the enclosure), digging through bedding, or moving throughout the enclosure were said to be exploring the environment.

During scoring an unanticipated behavior was noted. Rats were moving the object an unprecedented amount and in a way that was categorically different from how object exploration has been defined. Instead of sniffing, licking, or biting objects, rats were lifting them off the bottom of the enclosure, flipping them upside down, and running across the enclosure with it in their mouth. For simplicity’s sake, we elected to call this heightened degree of object manipulation object play behavior. Object play was defined as a subset of object exploration in which rats move the object. For a behavior to count as object play a rat must be attending to the object (snout orienting less than 2 cm) and in some way move the object. For example, rolling the object upside down, running across the enclosure with the object in their mouth, and rotating the object using forepaws or mouth were all included as object play behavior. Sniffing or biting the object without movement or any object movement caused by back paws or tail were not considered object play behavior. Our definition of object play in rats aligns closely with how object play is defined for use in Avian studies (see O’Hara and Auersperg, 2017). Since object play behavior was defined as a subset of object exploration inclusion of object play does not affect the ratio of context to object exploration. While many measures of play behaviors involve scoring interactions between conspecifics (Pellis and Pellis, 1998; Whishaw and Kolb, 2020), in the current study object play was determined by interactions between the rat and the object.

All videos were scored using BORIS (Friard and Gamba, 2016). The time index that the subject began and ceased demonstrating a behavior was recorded. The total duration of each behavior was calculated by subtracting the start times from stop times and adding each instance of that type of behavior together. Scorers were blind to transportation and object conditions during scoring.

### Procedure

#### Short Retention Interval

The short retention interval study utilized light objects in a modified novel object paradigm in a 2 × 2 between-subjects factorial design. Rats were habituated to the familiar object alone in the homecage for 10 min for 2 days. There were two levels of the object factor: familiar or novel. There were two levels of the transportation factor: familiar and novel. Rats were randomly assigned to one of these four groups.

The Novel Transportation procedure takes 2 days to run, after 3 days of handling to habituate rats to human contact. Familiarization to the familiar object takes place in two familiarization sessions. Both cage mates were removed from their homecage and placed into a rest cage and transported to a dark holding room for 5 min. One of two objects was chosen to become familiar and placed in the homecage (counterbalanced). The first rat was removed from the rest cage and placed in a transportation cage to be re-introduced to the homecage with the object. The first rat was allowed to explore the homecage and object for 10 min while their cage mate remained in the rest cage. The first rat was then removed and returned to the holding room. The object was switched out for the familiar object designated for the second rat. The second rat was transported back to the homecage for their 10-min exploration session. The second rat was removed from the homecage and reunited with its cage mate in the rest cage in the holding room before both rats are returned to their empty homecage. All transportation during familiarization was identical across all subjects. This procedure was repeated on day 2.

Testing occurred 1 h after the second familiarization session. Both rats were removed from their homecage and put into a rest cage and rest in the holding room for 5 min. It is at this point that some pairs of animals experienced novel transportation. Either the familiar or a novel object was placed in the testing environment. The first rat was then placed in a transportation cage and moved to the testing environment and recorded for 3 min. The first rat was returned to the holding room after testing and the second rat was removed and tested in the same way as the first rat. After the second rat had finished testing both rats were placed in the same rest cage and returned to the homecage.

#### Long Retention Interval

The long retention interval study utilizes the same Novel Transportation paradigm with few alterations. This study employed a 2 × 3 between-subjects factorial design. Rats habituated to the light familiar object alone in the homecage for 10 min for 2 days. There were two levels of the object factor: familiar or novel. There were three levels of the context factor: familiar (homecage), novel (operant box), and novel transportation (novel transportation to operant box). Rats were randomly assigned to one of six groups.

The long retention interval procedure takes 3 days to run, after 3 days of handling to habituate rats to human contact. The first 2 days of the procedure are object familiarization. The third day is the testing day. One-third of the rats were transported using novel cues on this day. Either the familiar or a novel object was placed in the testing environment (the homecage is the familiar environment, a standard operant box is the novel environment, and the novel transportation context is the same operant box but with different transportation cues).

#### Anchored Object

In order to minimize potential confounds from object play, heavy objects were used to ensure that rats could not displace them in this study. Otherwise, the anchored object experiment was conducted identically to the long retention interval study.

## Results

R Studio was used to analyze the data. For the short retention interval study, a 2 × 2 object (familiar and novel) × transportation (familiar and novel) between-subject’s analysis of variance (ANOVA) was conducted using the total duration of object exploration data. For the long retention interval study, a 2 × 3 object (familiar and novel) × context (familiar, novel, and novel transportation) between-subject’s ANOVA was conducted using the total duration of object exploration data. This analysis was repeated using the total duration of object play behavior. The anchored object study also utilized a 2 × 3 object (familiar and novel) × context (familiar, novel, and novel transportation) between-subject’s ANOVA to test the total duration of object exploration. Tukey’s HSD tests were conducted *Post hoc* as necessary. All error values reported represent the standard error of the mean (SEM). See Supplementary Materials for additional analyses.

### Short Retention Interval

This study demonstrates that transportation cues may be a part of a broader understanding of context. The total duration of object exploration differed as a function of object familiarity (*F*_(1, 21)_ = 6.15, *p* = 0.022). Rats spend more time with novel objects (*M* = 74.5 ± 14.2 s) compared to familiar (*M* = 35.9 ± 8.2 s). There is a trend to suggest that object exploration also depends on transportation familiarity (*F*_(1, 21)_ = 3.41, *p* = 0.071). Rats spend less time with the object after novel transportation (*M* = 40.8 ± 9.0 s) compared to familiar (*M* = 69.6 ± 14.8 s). There was no interaction between object and transportation familiarity (*F*_(1, 20)_ = 1.308, *p* = 0.27). While the effect of transportation was only marginally significant, its effect size was large (partial *η*^2^ = 0.14) and the relatively small group sizes (*n* = 6) contributed to the study’s low power [(1− β) = 0.47]. Additionally, novelty-induced differences in exploration were demonstrated between subjects for the first time. The increased behavioral variability of a between-subjects design may have also obfuscated results.

**Figure 3:**
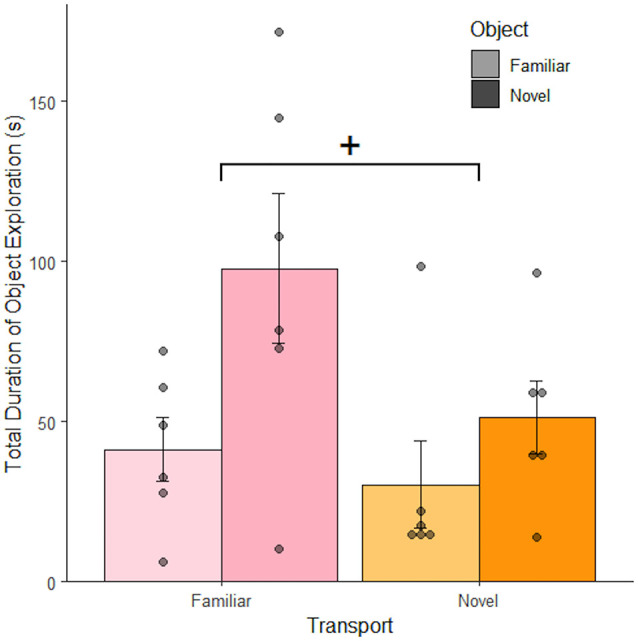
Object exploration increased with novel objects and familiar transportation. Rats exposed to a novel object spent more time exploring it than rats exposed to a familiar object 1 h after the final object familiarization session. The introduction of novel transportation cues lead to a decrease in the mean total amount of time rats spent exploring the object; demonstrating that they were instead exploring the context (*n* = 24). Error bars report SEM for each group. Points indicate the total duration of object exploration for each subject grouped into bins of 5.5 s. ^+^*p* < 0.1.

Since environment exploration was calculated by subtracting object exploration and non-exploratory behaviors from the total duration of the observation, exploration of the environment and the object are mutually exclusive. As such, these data reflect increased interest in context as object exploration decreases. Therefore, context exploration increased with novel transportation (Figure 3), suggesting that transportation cues impact perceived context. We next asked if the effects of transportation on context perception were robust enough to remain with a greater recall delay.

### Long Retention Interval

**Figure 4:**
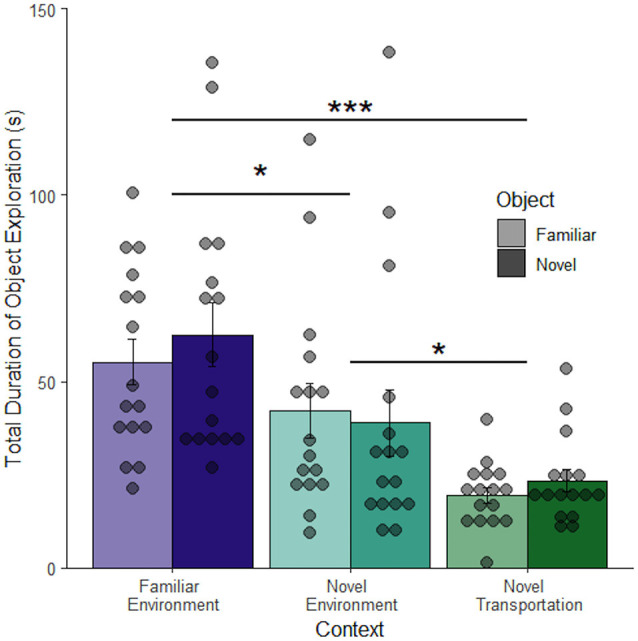
Transportation but not object effects are retained in a novel environment. When the retention period was extended to 24 h there was no difference between mean exploration of a familiar compared to a novel object. Novel transportation to a novel environment leads to a decrease in mean object exploration, and therefore an increase in context exploration. There was a slight difference in exploration in familiar and novel environments proceeded by familiar transportation cues (*n* = 96). Error bars report SEM for each group. Points indicate the total duration of object exploration for each subject grouped into bins of 4.6 s. ***p < 0.001, *p < 0.05.

Figure 4 shows that duration of object exploration differs depending on context (*F*_(2, 92)_ = 16.70, *p* < 0.001), but not object (*F*_(1, 92)_ = 0.26, *p* = 0.61) and no interaction between context and object (*F*_(2, 90)_ = 0.35, *p* = 0.71) 24 h after object familiarization (Figure 4). Rats spent significantly less time exploring the object after novel transportation (*M* = 21.39 ± 3.94 s) than after familiar transportation to the familiar context (*M* = 58.87 ± 0.31 s, adjusted *p* < 0.001) and novel context (*M* = 40.52 ± 0.86 s, adjusted *p* = 0.011). There was also a difference between familiar and novel contexts (adjusted *p* = 0.016). This shows that novel transportation leads to increased context exploration. Since context exploration increased in the same novel environment with the addition of novel transport, novel transport may increase the perceived novelty of a context. Since the familiar and novel contexts were completely separate spaces in this study, these results show that relative exploration of either an object or the environment is an effective measure to compare rodent behavior across contexts. The lack of difference between the novel and familiar objects in the familiar environment contradicts what was expected based on the novel object literature (Dix and Aggleton, 1999; Antunes and Biala, 2012). However, the novel object novel context procedure used here differs considerably from traditional novel object paradigms. In this study, object exploration is compared to environment exploration rather than interaction with another object.

**Figure 5:**
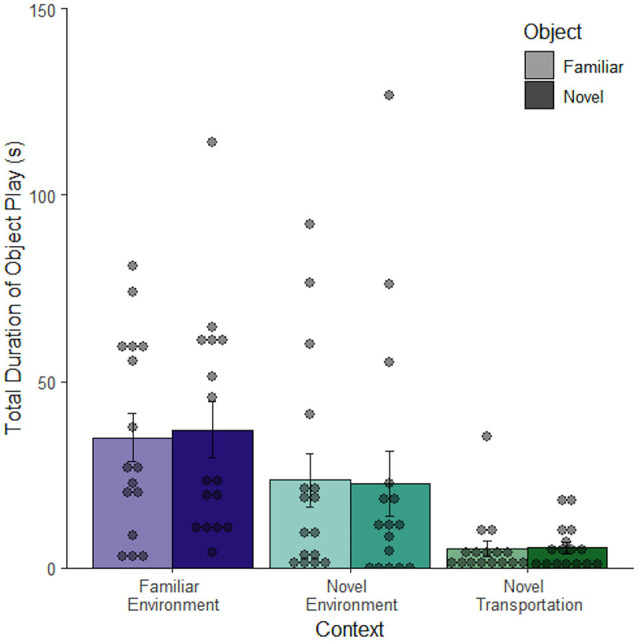
Time spent playing with an object changes depending on context. Rats in a familiar context spend more time playing with an object than rats in a novel context. Novel transportation is also related to decreased mean object play (*n* = 96). Error bars report SEM for each group. Points indicate the total duration of object exploration for each subject grouped into bins of 4.2 s.

Intentional movement or manipulation of the object was deemed to be object play behavior, which was considered a subset of object exploration. Object play behavior changed depending on context (*F*_(2, 92)_ = 12.43, *p* < 0.001) with the most object play occurring in the familiar context (*M* = 35.94 ± 4.92 s), less in the novel context (*M* = 23.13 ± 5.57 s), and even less in the novel context after novel transportation (*M* = 5.21 ± 1.30 s). In the novel transportation context, less than half of the rats exhibited any object play behavior, while every rat played with the object at least once in the familiar environment (Figure 5). Since object play differed across contexts it may have confounded the relative exploration of objects in different contexts. In order to eliminate this confound, the next study utilized immobile objects aimed at reducing object play behavior.

### Anchored Object

**Figure 6:**
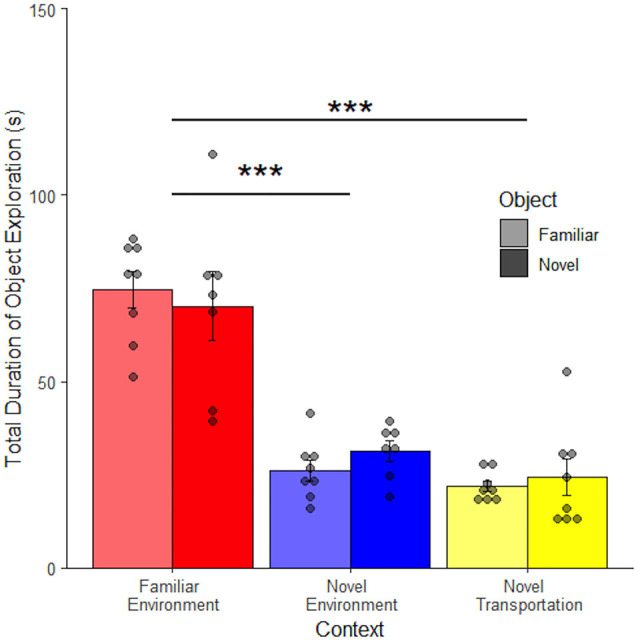
Effects of novel transportation dissipate when an anchored object is introduced. There is no difference in the mean duration of object exploration between the novel and familiar objects or contexts with changes in transportation cues when object play is eliminated *via* anchoring of the object (*n* = 46). Error bars report SEM for each group. Points indicate the total duration of object exploration for each subject grouped into bins of 3.3 s. ***p < 0.001.

The anchored object long retention interval study showed a similar pattern of results as the long retention interval study (Figure 6): there were clear effects of context but not object for every measure of exploration. The total duration of anchored object exploration was related to context familiarity (*F*_(2, 42)_ = 65.25, *p* < 0.001), but not object familiarity (*F*_(1, 42)_ = 0.003, *p* = 0.96) and no interaction between context and object (*F*_(2, 40)_ = 0.51, *p* = 0.60). There was not a significant difference between familiar transportation (*M* = 28.58 ± 1.99 s) and novel transportation (*M* = 23.05 ± 2.48 s, adjusted *p* = 0.47) to a novel context. However, rats spent more time exploring an object in a familiar environment (*M* = 72.57 ± 4.84 s) compared to novel independent of transportation (Familiar Transportation: adjusted *p* < 0.001, Novel Transportation: adjusted *p* < 0.001). The use of a heavy object to restrict object play behavior was effective since 0 s of object play were recorded during the second study. The total duration of non-exploratory behaviors also decreased; no non-exploratory behaviors occurred in the novel environment (Supplementary Figure 3).

## Discussion

### Transportation Impacts Context

The three studies presented here were an investigation into multi-modal factors influencing novelty-induced exploration indicative of context-dependent memory in rats. In the novel transportation paradigm, a single object is placed in an environment as a way to indirectly measure context exploration: a rat is exploring a context if it is not exploring the object (or engaged in non-exploratory activity like grooming). Therefore, a decrease in object exploration indicates an increase in context exploration. The short retention interval study showed a trend indicating that object exploration changed after rats were transported *via* novel means: object exploration decreased after novel transportation and novelty-induced exploration of the novel object decreased relative to familiar transportation (Figure 3). These data suggest that transportation cues can lead to novelty-induced exploration of a context; indicating that transportation cues influence a rat’s perception of context. Previous studies have shown that novel contexts can disrupt memory as demonstrated by decreased exploration of a novel object relative to a familiar one (Wilson et al., 2013; Arias et al., 2015). This suggests that transportation cues are part of a larger context that extends beyond the testing environment. Bevins et al. (2000) showed that rats conditioned, at least in part, to the transportation cues preceding entrance to an environment. The current studies extend upon these findings by showing that transportation cues are likely perceived as part of a context and are used in rats’ broader contextual perception beyond predicting shock. The long retention interval study showed that transportation’s effects of context remain when the delay between object familiarization and testing is increased to 24 h and in a novel environment. However, the anchored object study showed that when a rat’s ability to play with the object is removed the effects of novel transportation disappear. It is unclear whether this is due to the object play itself, the decreased behavioral range, or the decreased saliency of the object within the context. A major limitation of these data is the lack of data on novel transportation preceding a familiar environment with a long retention interval. This makes some of our comparisons between studies indirect and it is, therefore, difficult to separate effects caused by transportation-altered contexts and novelty-induced stress.

### Modulations of Transportation Effects

Both studies that utilized a 24-h retention period (Long retention interval and Anchored Object) showed a similar pattern of results: object exploration changed depending on the environment, but not object, novelty. In the long retention interval study, which used light objects that the rats were able to play with, there were significant differences between the novel transportation and novel environment contexts indicating that the novel transportation cues presented in the novel transportation condition impacted how the rats interacted with the context. However, in the anchored object study objects were immobile and there was not a significant difference between the novel and novel transportation groups. Additionally, the tightened error in the anchored object study suggests that reducing object play also reduced the behavioral range available (Figure 6). Reduced behavioral range may also be related to reduced non-exploratory behaviors. A non-zero amount of non-exploratory behaviors was recorded in the second study; however, these behaviors were only recorded in the familiar environment (Supplementary Figure 3). The differences in behavior between the long retention interval study to the anchored object suggest that the removal of the object play variable influenced how the rats interacted with their environment. Object play is more typically measured as an interaction between rats (see Whishaw and Kolb, 2020). Future studies should address object play with both familiar and novel conspecifics to test the implications for play discussed in the present studies.

In the long retention interval study, object exploration showed a clear difference between novel and novel transportation contexts suggesting that transportation cues play an important role in how rats distinguish between contexts. However, this result was not reliably replicated in the anchored object study. This difference could be related to a smaller sample size in the anchored object study, but when collapsing by context the power is still sufficient to expect meaningful results. There is a possible ceiling effect when analyzing environment exploration; however, there is still no difference between novel and novel transportation contexts when looking at object exploration which does not suffer either ceiling or floor effects. Given the inhibited behavioral range and lower variability in the anchored object study, effects should be easier to detect, not harder. Therefore, the difference in context differentiation between the two studies is likely related to the ability to play in the long retention interval study but not in the anchored object study.

The heavier objects used in the anchored object study did not allow for rats to play with them as they tended to in the first two studies. This difference in object interaction may have led to differences in stimulus encoding. Less-salient cues can ‘fade into the background’ and become a feature of the environment rather than an independent cue (Nadel and Willner, 1980). Since rats could not interact with the anchored objects in the same way they may have been less salient and likely faded from the foreground in the first two studies and into the background and were encoded as a contextual cue rather than an independent cue in the anchored object study. The presence of an object distinct from the context is integral to the design of the current studies; without the addition of an object, there is no clear way to quantify context exploration. Do anchored objects have enough salience to be used in contrast to the surrounding context? While object play behavior was eliminated in the anchored object study, the mean total duration of exploration remained similar to the previous two studies. This suggests that rats distinguished between anchored objects and the surrounding context and that the logic of comparing relative exploration of the object and the context should still hold. Future work should clarify how cues and cue salience interact with the encoding of an environmental and contextual recall. However, differences in salience do not fully explain the differences in exploration between the familiar and novel transportation contexts seen in the first two studies.

Object play may affect both object and environment exploration behavior due to its relationship with stress. In the novel transportation paradigm rats are exposed to an operant chamber for the first time in the testing phase. This may cause greater stress compared to studies where rats have habituated to behavioral apparatuses as it is well known that novelty induces stress in rodents (Bassett and Cairncross, 1973; Baldwin et al., 1974). Additionally, introduction to the operant chamber during the test period in these studies may be more stressful than typical manipulations of context novelty in which rats are allowed to habituate to a behavioral apparatus as a familiar context. The rats in this study may have failed to successfully differentiate between familiar and novel objects after 24 h if they experienced increased stress. More work is needed to compare manipulations both within the same space and between distinct places across differing levels of novelty. Since stress has been known to impair both novel object recognition (Eagle et al., 2013) and contextual fear conditioning (Cordero et al., 2003), novelty-induced stress may have differentially impacted groups in the current studies. If rats that were able to reduce their stress by playing (Arelis, 2006), they may be better able to distinguish novel objects and contexts. Additionally, interoceptive cues related to stress have been shown to impact reinstatement of extinguished behavior (Schepers and Bouton, 2019). Stress may create a distinct set of interoceptive cues and therefore represent another contextual modality which influences behavior. A More direct measures of interoceptive cues related to play and stress is needed to understand the relationship between internal states and perceived context how interoceptive cues interact with exteroceptive cues. In the current study, the least amount of object play occurred in the most novel (novel transportation) context which presumably contains the greatest number of novel cues and therefore should elicit the most novelty-induced stress. The effects of play on behavioral variability, stress, and novelty-induced exploration of objects and environments need to be systematically tested in future experiments further refined.

The long retention interval study expanded upon the trend indicating novel transportation impacts perceived context from the short retention interval study, in a novel environment and with a longer retention period (Figure 4). However, rats did not favor exploring novel objects with the longer retention period between object familiarization and testing. The lack of object differences was recapitulated in the anchored object study. Since the short retention interval study analyzed only familiar environments, it is therefore unclear whether increased stress from novel environments or the longer retention period accounts for the lack of object differentiation seen in the long retention interval and anchored object studies. While the novel transportation protocol used differs substantially from novel object recognition, differences between the novel and familiar objects were anticipated. The long retention interval and anchored object studies utilized a relatively long wait between object familiarization and recall (24 h) which, while common in the literature (see Antunes and Biala, 2012), may make the task prohibitively difficult and obscure results. Given this limitation of the current data it is unclear whether novel transportation to different environments has a differential impact on memory and behavior. Given the lack of object differentiation in familiar environments and what we know about object differentiation from other paradigms, it is likely that the retention period negatively impacted object differentiation (Ennaceur and Delacour, 1988; Antunes and Biala, 2012). More work is needed to determine the threshold retention period and whether additional factors such as sleep or consolidation impact object memory in this paradigm.

### Conclusions

In this study, we sought to examine the effects of novel transportation cues on rats’ experience of context. The Novel Transportation procedure, derived from Novel Object Recognition tasks, allowed us to quantify exploration of an environment relative to an object within the environment. Novel transportation led to increased context exploration and decreased differentiation between novel and familiar objects. These data suggest that novel transportation cues can lead to novelty-induced context exploration. Therefore, transportation cues influence a rat’s perception of context and may also be a part of a larger context that extends beyond the testing environment. Transportation’s effect on context may be moderated by retention period, the familiarity of testing environment, and behavioral range (i.e., ability to play with an object). These data highlight the importance of defining context broadly in behavioral science and suggest that future experimental manipulations of context should include transportation.

## Data Availability Statement

The datasets presented in this study can be found in online repositories. The names of the repository/repositories and accession number(s) can be found below: https://doi.org/10.18738/T8/GVT0EE.

## Ethics Statement

The animal study was reviewed and approved by IACUC at the University of Texas at Austin.

## Author Contributions

MM and VN designed the study. VN ran the study, analyzed the data, wrote the first draft of the manuscript, and approved the final version of the manuscript. MM helped with data analysis and interpretation, edited the manuscript, and approved the final version of the manuscript. SS, CM, LA, and MR helped with data interpretation, helped with running the experiments, and approved the final version of the manuscript. All authors contributed to the article and approved the submitted version.

## Conflict of Interest

The authors declare that the research was conducted in the absence of any commercial or financial relationships that could be construed as a potential conflict of interest.

## Publisher’s Note

All claims expressed in this article are solely those of the authors and do not necessarily represent those of their affiliated organizations, or those of the publisher, the editors and the reviewers. Any product that may be evaluated in this article, or claim that may be made by its manufacturer, is not guaranteed or endorsed by the publisher.
