# Supplementary material for: Altering Perceived Context: Transportation Cues Influence Novelty-Induced Context Exploration
Source: Front Behav Neurosci. 2021 Jul 29;15:714927. doi: 10.3389/fnbeh.2021.714927 (PMC8358674; doi:10.3389/fnbeh.2021.714927)
Supplement: Supplementary file 1 [file Data_Sheet_1.docx]

**Additional Statistics**

Each dataset was tested to see if it met the assumptions of analysis of variance (ANOVA) testing. Normalcy of residuals was tested visually using qq-plots and quantitatively using the Shapiro-Wilk normality test. Homogeneity of variance was tested visually using boxplots and quantitatively using the Breusch-Pagan test. Independence was assumed based on random assignment to groups. The short retention interval data did not demonstrate normalcy of residuals (W = 0.94, p = 0.14). This data technically passed the homogeneity of variance assumption (F_(1,22)_=4.14, p=0.23), but visual examination did not suggest homogeneous variance. The long retention interval data failed to meet the assumptions of normalcy of residuals (W = 0.89, p <0.001) and homogeneity of variance (F_(2,93)_=4.14, p=0.019). The anchored object data similarly failed the normalcy of residuals (W = 0.94, p= 0.023) and homogeneity of variance (F_(2,43)_=3.69, p=0.033) assumptions. Since none of the data met all of the assumptions of analysis of variance (ANOVA) testing, simulation techniques were employed. For each experiment, a Monte Carlo style simulation randomly scrambled the original data to produce a dataset in which there is no relationship between outcome variables and object, transport, and context conditions. 10, 000 iterations were used for each simulation. ANOVAs were performed on the simulated data to generate a comparison distribution of F values reflecting no systematic relationship in the data. The F value from the original ANOVA was then evaluated against the comparison distribution and a p value was calculated by determining the number of iterations in which the F value from the randomly generated data exceeded the original F value, divided by the total number of iterations. As supplementary table 1 shows, the simulated p values were nearly identical to the traditional statistical values; in no case did use of the simulation alter the interpretation of the results. This lack of difference between the two techniques suggests that even though the data failed to meet all the assumptions of ANOVA testing, they followed the theoretical distributions closely enough that the results of the traditional test are accurate.

**Supplementary Table 1. Probability of rejecting the null hypothesis remains similar across methods of statistical testing.** The same F values were used for both methods. Traditional ANOVA p values were taken from summary ANOVA tables. Monte Carlo simulation p values were calculated by determining the probability of achieving an F value as high as the value from the original ANOVA.

|  | Statistical Value | Traditional ANOVA | Monte Carlo Simulation |
| --- | --- | --- | --- |
| Short Retention Interval  Object by Transportation Interaction | F_1, 20_=1.308 | p=0.2663 | p=0.2667 |
| Short Retention Interval  Object Main Effect | F_1, 21_=6.147 | p=0.0217 | p=0.0189 |
| Short Retention Interval Transportation Main Effect | F_1, 21_=3.407 | p=0.0791 | p=0.0778 |
| Long Retention Interval  Object by Context Interaction | F_2, 90_=0.350 | p=0.707 | p=0.7078 |
| Long Retention Interval  Object Main Effect | F_1, 92_=0.262 | p=0.61 | p=0.6178 |
| Long Retention Interval  Context Main Effect | F_2, 92_=16.697 | p= 6.51e-07 | p<0.001 |
| Long Retention Interval  Main Effect of Context on Object Play | F_2, 92_=12.427 | p=1.67e-05 | p<0.001 |
| Anchored Object  Object by Context Interaction | F_2, 40_=0.511 | p=0.604 | p=0.599 |
| Anchored Object  Object Main Effect | F_1, 42_=0.003 | p=0.96 | p=0.958 |
| Anchored Object  Context Main Effect | F_2, 42_=65.252 | p= 1.3e-13 | p<0.001 |

**Supplementary Figure 1. Number of seconds spent in various exploration activities in the short retention interval experiment.** The total duration of the test session was 3 minutes (180 seconds). Context exploration was defined as exploratory behavior that does not meet the criterion for object exploration and was calculated by subtracting duration of object exploration and non-exploratory behaviors from the duration of the observation period. Object play was defined as a subset of object exploration and is not shown here.

**Supplementary Figure 2. Number of seconds spent in various exploration activities in the long retention interval experiment.** The total duration of the test session was 3 minutes (180 seconds). Context exploration was defined as exploratory behavior that does not meet the criterion for object exploration and was calculated by subtracting duration of object exploration and non-exploratory behaviors from the duration of the observation period. Object play was defined as a subset of object exploration and is not shown here.

**Supplementary Figure 3. Number of seconds spent in various exploration activities in the anchored object experiment.** The total duration of the test session was 3 minutes (180 seconds). Context exploration was defined as exploratory behavior that does not meet the criterion for object exploration and was calculated by subtracting duration of object exploration and non-exploratory behaviors from the duration of the observation period. Object play was defined as a subset of object exploration and is not shown here.
