# Supplementary figures and images for: Altering Perceived Context: Transportation Cues Influence Novelty-Induced Context Exploration
Source: Front Behav Neurosci. 2021 Jul 29;15:714927. doi: 10.3389/fnbeh.2021.714927 (PMC8358674; doi:10.3389/fnbeh.2021.714927)

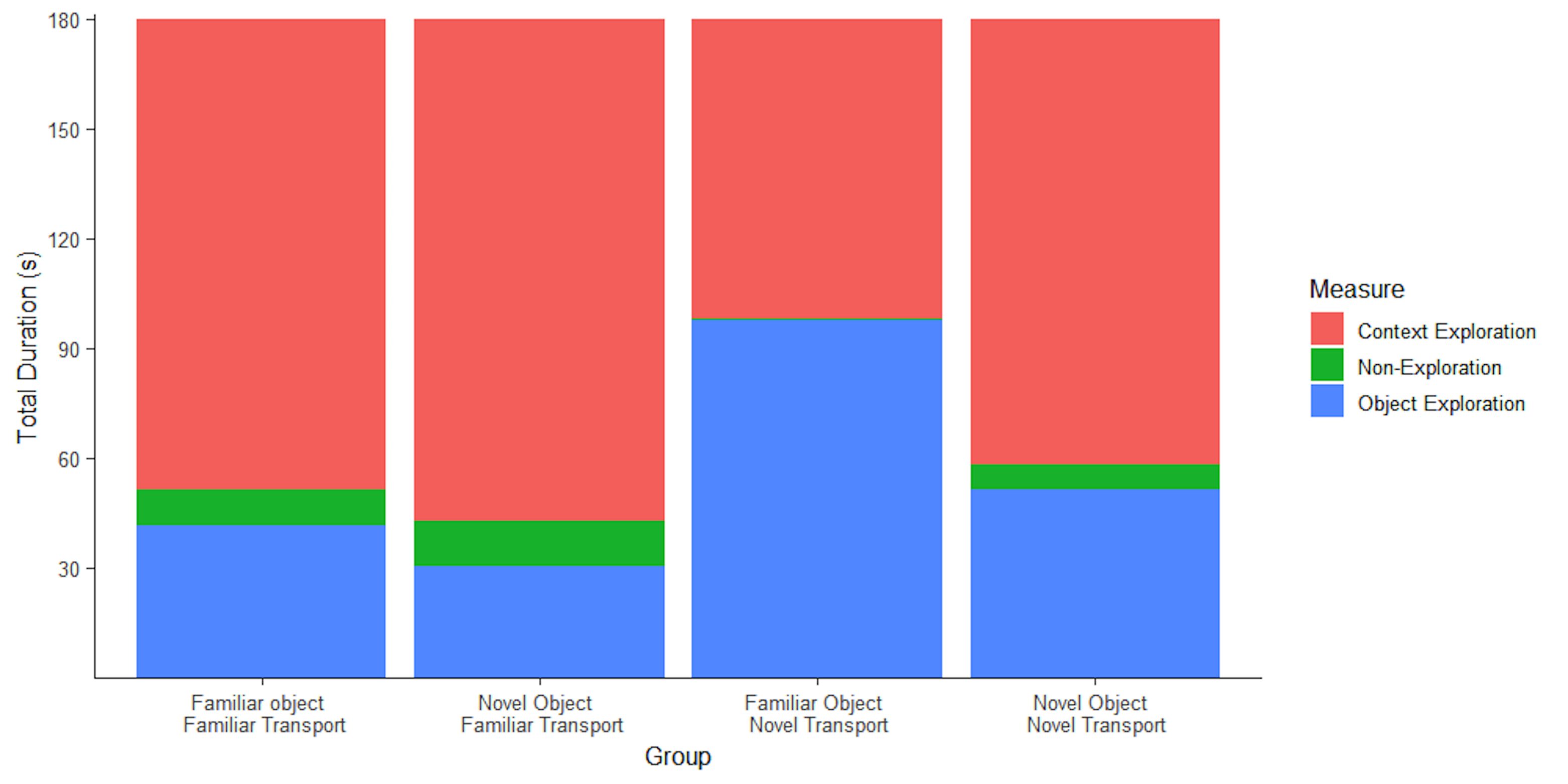

Supplement: Supplementary file 2 [file Image_1.tiff]

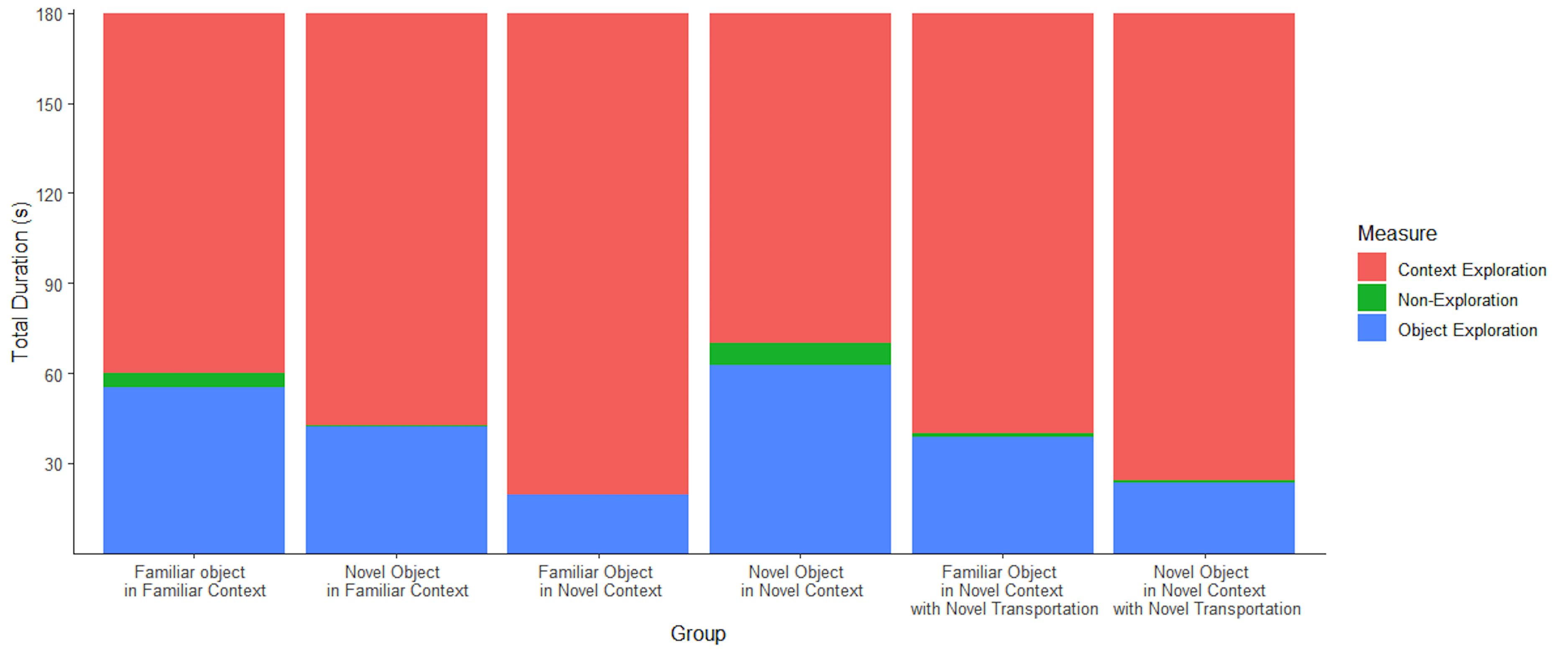

Supplement: Supplementary file 3 [file Image_2.tiff]

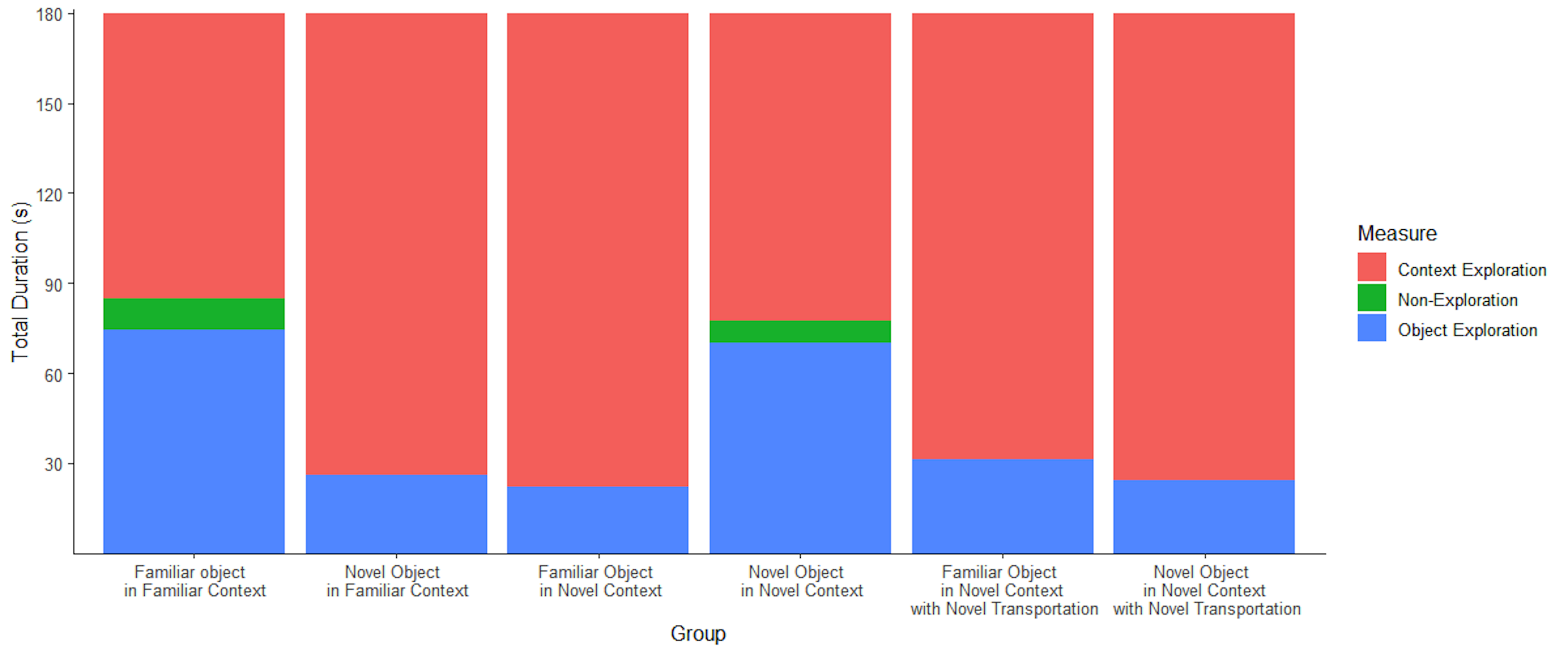

Supplement: Supplementary file 4 [file Image_3.tiff]
